# Case report: Magnesium—A new therapeutic target in gestational diabetes mellitus?

**DOI:** 10.1002/ccr3.3309

**Published:** 2020-09-07

**Authors:** Kun Zhang

**Affiliations:** ^1^ Department of Internal Medicine and Cardiology Charité – Universitätsmedizin Berlin Germany; ^2^ Berlin Health Institute Berlin Germany

**Keywords:** fasting glucose, gestational diabetes mellitus, insulin, magnesium

## Abstract

To date, physicians are not aware of a potential connection between magnesium and gestational diabetes mellitus. This case shows that magnesium supplementation can reduce maternal serum glucose, specifically the hard‐to‐control fasting glucose.

## Graphical abstract

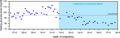

## 1 INTRODUCTION

Gestational diabetes mellitus (GDM) is a growing health concern in many parts of the world and has gained increasing attention in the past years. It is defined as impaired glucose metabolism in pregnant women who did not suffer from diabetes beforehand with an estimated prevalence of up to 28% in some regions.^1^ Sex hormones, that is estrogen, progesterone, and prolactin, are assumed to be responsible for the increased insulin resistance and impaired glucose tolerance during pregnancy.^2^ GDM has serious short‐ and long‐term consequences, including higher risk for preeclampsia, caesarian section and development of diabetes mellitus type 2 (DM II) for the mother and large for gestational age (LGA), postnatal hypoglycemia and hyperbilirubinemia as well as higher risk for DM II later in life for the offspring.^1^, ^2^ Insulin is administered as a therapeutic measure when dietary and lifestyle changes are insufficient to improve glucose metabolism. However, as simple as it may seem, application of insulin s.c. can be associated with a number of negative aspects, for example regarding its handling, risk for hypoglycemia, and impact on birth‐giving procedure as well as subsequent psychological issues for the mother.

This case report shows that oral supplementation of magnesium (Mg) can be an effective therapy in gestational diabetes.

## 2 CASE PRESENTATION

Here, we report on a 36‐year‐old woman, 2 gravida 1 para, BMI 21 kg/m^2^ who has been diagnosed with GDM in week 24 + 2. During her first pregnancy, she was diagnosed with GDM in week 24 + 6 which was successfully controlled with dietary adjustments and no insulin therapy. She gave birth to a healthy male infant with normal weight and no postnatal complications upon spontaneous delivery in week 41 + 1. In her previous medical history, she had a laparoscopic appendectomy and hemithyroidectomy with stable daily L‐thyroxine 50 µg intake. TSH was regularly checked. Currently, the patient has, in addition, been taking supplementary folic acid 400 µg plus vitamin D 800 IE p.o.. She has no family history of DM II and/or GDM. No other chronic diseases are known.

**FIGURE 1:**
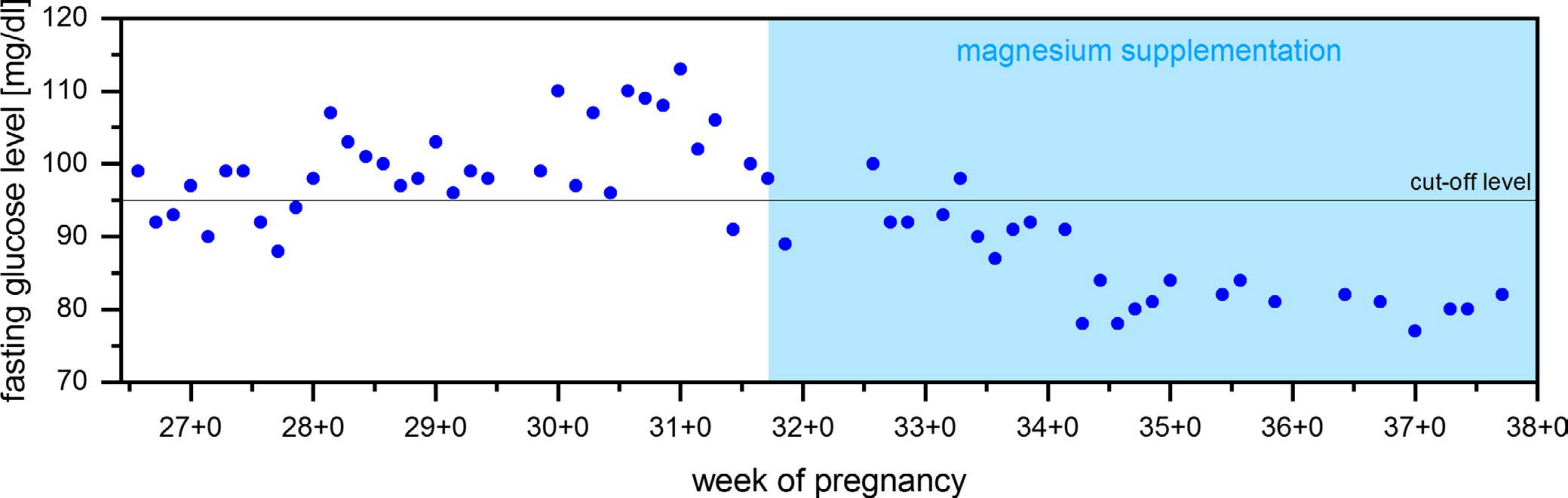
Fasting glucose levels showing a steady increase over time. After starting magnesium supplementation, fasting glucose levels are falling back into therapeutic range.

Due to the experiences of the first pregnancy, a change in diet and lifestyle was already conducted from the beginning of the second pregnancy. Nevertheless, the 75 g oral glucose tolerance test was positive for GDM, showing following intravenous values: 88 mg/dL baseline, 184 mg/dL after 1 hour, and 150 mg/dL after 2 hours (normal range: <92 mg/dL, <180 mg/dL, <153 mg/dL). After diagnosis of GDM, the patient consequently measured capillary blood sugar levels four times a day with a calibrated blood glucose meter (Acsensia Contour Next, Germany): morning fasting glucose and one hour after breakfast, lunch, and dinner. Dietary changes included decreased intake of fast‐acting carbohydrates and splitting meals into six a day. Moreover, the patient exercised 30 minutes per day using an ergometer. Gain of weight during pregnancy was optimal with approximately 8 kg in 30 weeks. While glucose levels remained within limits during the day, the morning fasting glucose was high and an increasing tendency was noticed (fasting glucose 100 ± 6 mg/dL, n = 32; 1 hour after breakfast 116 ± 19 mg/dL, n = 22; 1 hour after lunch 122 ± 20 mg/dL, n = 16; 1 hour after dinner 111 ± 15 mg/dL, n = 14). Sonographic fetal biometric measurements showed normal development. However, more than 50% of the fasting glucose values were above the cut‐off value of 95 mg/dL with an increasing tendency over time and insulin therapy was taken into account (Figure 1).

Starting from week 31 + 0, the patient complained about calf cramps which occurred almost every morning. Thus, a supplementary therapy with Mg 200 mg per day was given. Serum Mg levels were within the normal range (1.8 mg/dL [normal range: 1.6‐2.5 mg/dL]). Calf cramps soon disappeared under treatment. Intriguingly, fasting glucose levels evidently decreased upon Mg treatment and remained under the cut‐off value 95 mg/dL (Figure 1). Insulin therapy could be avoided using Mg supplementation. At week 38 + 0, a healthy male infant (51 cm, 3250 g) was spontaneously delivered.

## 3 DISCUSSION

This case report shows in a pregnant woman with GDM that Mg supplementation can be an effective means to reduce maternal serum glucose levels and to prevent from insulin therapy. This is noteworthy specifically with respect to fasting glucose which can otherwise be difficult to control with dietary and lifestyle changes alone as was observed in this case.

Magnesium is essential for the body in multiple aspects, being a co‐factor for enzymes, regulating ion channels and maintaining cell homeostasis. Pregnant women are prone to Mg loss and, moreover, it has been described that specifically intracellular Mg depletion occurs in pregnancy.^3^ Studies showed that lack of Mg is associated with metabolic syndrome, insulin resistance, and/or DM II. On the molecular level, reduced intracellular Mg concentrations impair insulin receptor activity and post‐receptorial insulin action.^4^, ^5^, ^6^ Lack of intracellular Mg may be present without being overt in serum levels and thus remains undetectable in routine laboratory findings.^7^

Only two studies exist reporting on Mg and GDM. Asemi et al^8^ reported that supplementation of 250 mg Mg for 6 weeks resulted in lower maternal serum glucose levels, lower incidence of newborn hyperbilirubinemia, and newborn hospitalization rate. No significant differences in the cesarean delivery rate or the need for insulin therapy were observed. It is to be noted that the study cohort was small with 35 patients per group and only 2 out of 35 in the placebo group and 1 out of 35 in the intervention group required insulin therapy. In another small study with 30 patients per group, a magnesium‐zinc‐calcium‐vitamin D complex (with 200 mg Mg per day) was supplemented for 6 weeks in pregnant women with GDM and no insulin dependence, which showed a reduction in maternal serum glucose and decreased biomarkers of inflammation and oxidative stress.^9^

To date, physicians and obstetricians, even those specialized in GDM, are not aware of a potential connection between Mg and GDM. Studies on this subject are sparse, but evidence suggesting causality is accumulating, mainly based on studies with DM II patients.^10^, ^11^, ^12^ Questions regarding the optimal dosage, duration of treatment as well as a suitable control for Mg supplementation remain to be answered. Even a patient with normal serum magnesium levels can profit from magnesium supplementation as highlighted here. It is the intracellular magnesium that seems to be crucial as mentioned above.^3^, ^4^, ^5^, ^6^ Furthermore, it could be speculated if early Mg treatment may even prevent manifestation of GDM.

Current guidelines suggest the application of insulin when more than 50% of the fasting glucose values are above 95 mg/dL, disregarding other parameters.^13^ The use the oral antidiabetic metformin has gained importance in the past years.^14^ However, medication with metformin during pregnancy is controversial. The National Institute for Health and Clinical Excellence suggests metformin as the first‐line drug. In other countries—such as Germany—it is not approved and can be only applied off‐label under certain circumstances when insulin treatment may be problematic.^15^ Other oral antidiabetics such as sulfonylureas are contraindicated during pregnancy. In contrast to insulin or metformin, the advantages of oral magnesium supplementation are obvious—it is safe, simple to use, and inexpensive.

In the era of world‐wide increasing incidence of GDM with all its consequences, the potential improvement of glucose metabolism by something as convenient as oral Mg supplementation is a ray of hope. In order to further evaluate the usefulness of its application, larger randomized controlled studies in women with GDM are necessary.

## CONFLICT OF INTEREST

None.

## AUTHOR CONTRIBUTIONS

KZ: analyzed the data and wrote the manuscript.

## ETHICAL APPROVAL

Ethical approval was not necessary.

## CONSENT STATEMENT

Written consent was obtained from the patient. Proof of consent can be requested at any time.
